# Individual and County-Level Factors Associated with Use of Multiple Prescribers and Multiple Pharmacies to Obtain Opioid Prescriptions in California

**DOI:** 10.1371/journal.pone.0046246

**Published:** 2012-09-25

**Authors:** Huijun Han, Philip H. Kass, Barth L. Wilsey, Chin-Shang Li

**Affiliations:** 1 Department of Public Health Sciences, University of California Davis, Davis, California, United States of America; 2 Department of Population Health and Reproduction, University of California Davis, Davis, California, United States of America; 3 VA Northern California Health Care System and Department of Physical Medicine and Rehabilitation, University of California Davis Medical Center, Davis, California, United States of America; University of Louisville, United States of America

## Abstract

Use of multiple prescribers and pharmacies is a means by which some individuals misuse opioids. Community characteristics may be important determinants of the likelihood of this phenomenon independent of individual-level factors. This was a retrospective cohort study with individual-level data derived from California's statewide prescription drug monitoring program (PDMP) and county-level socioeconomic status (SES) data derived from the United States Census. Zero-truncated negative binomial (ZTNB) regression was used to model the association of individual factors (age, gender, drug schedule and drug dose type) and county SES factors (ethnicity, adult educational attainment, median household income, and physician availability) with the number of prescribers and the number of pharmacies that an individual used during a single year (2006). The incidence rates of new prescriber use and new pharmacy use for opioid prescriptions declined across increasing age groups. Males had a lower incidence rate of new prescriber use and new pharmacy use than females. The total number of licensed physicians and surgeons in a county was positively, linearly, and independently associated with the number of prescribers and pharmacies that individuals used for prescription opioids. In summary, younger age, female gender, and living in counties with more licensed physicians and surgeons were associated with use of more prescribers and/or more pharmacies for obtaining prescription opioids.

## Introduction

Acute and chronic pain affect over 100 million adults in the United States [1]. The annual economic cost associated with chronic pain is estimated at $560–635 billion [1]. Opioid analgesics have been acknowledged to be an effective treatment to control moderate to severe pain while simultaneously improving quality of life [2]. However, the abuse and diversion of these medications has led to a public health crisis. According to national surveillance reports in 2009, 35 million US residents aged 12 years or older reported non-medical use of opioids at least once during their lifetime [3]. The annual financial cost of prescription opioid abuse is estimated at about $10 billion [4]. The use of multiple prescribers (“doctor shopping”) is one of the most common methods that drug abusers and dealers employ to obtain prescription opioids for non-medical use [5], [6]. Identifying high-risk individuals for doctor shopping will play a significant role in controlling abuse and diversion of prescription opioids.

Illicit drug use and prescription opioid abuse tend to be intertwined [7]. The National Survey on Drug Use and Health (NSDUH) established that illicit drug use is associated with several individual socio-demographic factors. Specifically, illicit drug use is more common among young adults between 18 and 25 years of age, males, and high school dropouts [3], [8]. By contrast, prescription opioid abuse related to doctor shopping may be more consistent with the demographics of the chronic pain population. Such individuals are more likely to be middle-aged [9], female, and living below the poverty level [10]. Studying the demographics of individuals who utilize multiple prescribers will provide a better picture of who is using this approach to misuse prescribed opioids.

In recent years, geographic location and interrelated socioeconomic characteristics have been recognized as important factors that affect overall health. For instance, obesity, metabolic syndrome, cardiovascular disease, and cancer have all been associated with the socioeconomic environment independent of individual factors [11]–[16]. Residing in socially disadvantaged neighborhoods (characterized by low education attainment and poor economic status) was associated with higher exposure to cocaine in early adolescence [17], increased likelihood of adult drug use [18]–[20], or related hospitalization [21], and recidivism [22], [23]. Although these findings reinforce the importance of the neighborhood social context as a determinant of drug use, most of them come from studies using either a comprehensive summary index [17]–[19], [22] or a measure of only income or poverty [20], [21], [23] to represent the level of social economic disadvantage. Social epidemiologists have questioned this methodology and proposed examining specific neighborhood socioeconomic determinants instead of overall SES [24]. To our knowledge, no prior research has focused on neighborhood contextual factors associated with use of multiple prescribers and/or pharmacies for opioid prescriptions. The present study will examine the effect of both individual-level factors (age, gender, and Drug Enforcement Agency (DEA) scheduling and dose of opioid therapy) and county-based factors (ethnicity, education attainment, median household income, and physician availability) on use of multiple prescribers and/or pharmacies for prescription opioids.

## Results

### Descriptive statistics

There were 1,087,070 eligible individuals in the CURES database. However, socioeconomic information was not available for 18 California counties in US Census data, which led to an exclusion of 2.8% individuals. The final sample size was 1,057,012.

The number of distinct prescribers that an individual used in 2006 for prescription opioids had a mean of 2.09 and a range of 1 to 158. Approximately 51% (n = 536,408) of individuals obtained their prescriptions from one prescriber, 45% (n = 476,843) used two to five prescribers, and 4% (n = 43,761) used six or more prescribers. The number of pharmacies that an individual used in 2006 for prescription opioids had a mean of 1.75 and a range of 1 to 100. Approximately 59% of individuals used only one pharmacy (n = 623,357), and 39% (n = 411,704) used two to five pharmacies, 2% (n = 21,951) used six or more pharmacies in 2006.

Table 1 contains demographics of the study population. Notably, almost half (46.25%) were between 45 and 64 years of age, with the majority being female (62.15%). Most individuals receiving opioids (75.86%) utilized only Schedule III opioids, with the majority (84.38%) prescribed a low average daily dose of 40 mg or less of morphine-equivalents per day. There was considerable variation in the distribution of different neighborhood-level socio-economic measures among counties. For example, the median (range) was 3.5 (1.7 to 8.1) for percent of the population who were from multiple ethnic groups, 17 (7.9 to 37.7) for percent of adult residents who did not graduate from high school, $53,500 ($37,100 to $81,800) for median household income, and 3,309 (52 to 26,867) for the number of licensed physicians and surgeons in a county. Almost four-fifths (78.64%) of individuals receiving opioids lived in counties where less than 4% of the population were of two or more races. The majority (93%) of individuals receiving opioids lived in the 35 counties where less than 30 percent of residents 25 years of age or older did not graduate from high school. Three-fifths (60%) of individuals receiving opioids lived in counties where the median household income was less than $55,000. Almost two-thirds of individuals (64.47%) lived in 11 counties where at least 2,000 licensed physicians and surgeons were available.

**Table 1:** Characteristics of the study population from the CURES, 2006.

Factor	Overall individuals(N = 1,057,012)
Individual characteristics	
Age (years)	
18–34	12.49%
35–44	16.55%
45–64	46.25%
65–74	12.34%
75–100	12.37%
Gender	
Male	37.85%
Female	62.15%
Drug schedule	
only Schedule II	6.93%
only Schedule III	75.86%
both Schedule II and Schedule III	17.21%
Dose type	
≤40 mg/day	84.38%
>40 mg/day	15.62%
	
County characteristics	
Percent of multiracial population	
1–1.9	1.50%
2–2.9	34.72%
3–3.9	42.41%
4+	21.36%
Percent of residents who did not graduate from high school	
0.0–9.9	3.64%
10.0–19.9	49.34%
20.0–29.9	43.42%
30.0+	3.61%
Median annual household income ($10,000)	
3.5–4.4	14.65%
4.5–5.4	44.63%
5.5–6.4	17.37%
≥6.5	23.35%
Number of licensed physicians and surgeons	
<500	11.31%
500–999	12.13%
1000–1999	12.08%
≥2000	64.47%

### Factors associated with the incidence rate of prescriber use

The multivariable analyses showed that all factors were significantly (p<0.0001) associated with the incidence rate of new prescriber use (Tables 2 and 3). The incidence rate of new prescriber use for opioid prescriptions generally declined across increasing age groups, although the incidence rates were similar for the 18–34 and 35–44 year groups. Compared to the individuals aged 75 years or higher, individuals who were 18–34 years, 35–44 years, 45–64 years and 65–74 years had 2.13 (95% CI: 2.10, 2.15), 2.11 (95% CI: 2.09, 2.14), 1.69 (95% CI: 1.68, 1.71) and 1.23 (95% CI: 1.21, 1.24) times higher incidence rates of new prescriber use in one calendar year, respectively, when adjusting for other individual-level and county-based factors. Males had an 8% lower incidence rate of new prescriber use than females after adjusting for other factors (adjusted IRR = 0.92 (95% CI: 0.91, 0.92)). Individuals who used only Schedule III opioids had a higher incidence rate (adjusted IRR = 1.11 (95% CI: 1.10, 1.13)) of new prescriber use than those who solely used Schedule II opioids; individuals who used both Schedule II and III opioids had the highest incidence rate (adjusted IRR = 3.02 (95% CI: 2.98, 3.06)). Individuals who received high dose opioid therapy had an adjusted IRR of 1.38 (95% CI: 1.36, 1.39).

**Table 2:** Adjusted incidence rate ratios and 95% confidence intervals (CI) from multivariable ZTNB regression model of individual characteristics for prescriber use to obtain prescription opioids in California in 2006.

Factor	Adjusted IRR (95% CI)	P-value*
Age (years)		<0.0001
18–34	2.13 (2.10, 2.15)	
35–44	2.11 (2.09, 2.14)	
45–64	1.69 (1.68, 1.71)	
65–74	1.23 (1.21, 1.24)	
75–100	1.00	
Gender		<0.0001
Male	0.92 (0.91, 0.92)	
Female	1.00	
Drug schedule		<0.0001
only Schedule II	1.00	
only Schedule III	1.11 (1.10, 1.13)	
both Schedule II and Schedule III	3.02 (2.98, 3.06)	
Dose type		<0.0001
≤40 mg/day	1.00	
>40 mg/day	1.38 (1.36, 1.39)	

Note. Factors in the table adjusted each other through this multivariable ZTNB regression model.

* p-value from F-test for assessing the effect of a factor in multivariable analysis.

**Table 3:** Adjusted incidence rate ratios and 95% confidence intervals (CI) from multivariable ZTNB regression model of county characteristics for prescriber use to obtain prescription opioids in California in 2006.

Factor	Adjusted IRR (95% CI)	P-value*
Percent of multiracial population		<0.0001
1–1.9	0.92 (0.89, 0.95)	
2–2.9	0.96 (0.95, 0.97)	
3–3.9	0.96 (0.95, 0.96)	
4+	1.00	
Percent of residents who did not graduate from high school		<0.0001
0.0–9.9	0.92 (0.89, 0.94)	
10.0–19.9	0.92 (0.90, 0.95)	
20.0–29.9	0.92 (0.90, 0.94)	
30.0+	1.00	
Median annual household income		<0.0001
35,000–44,999	0.98 (0.96, 0.99)	
45,000–54,999	0.97 (0.96, 0.98)	
55,000–64,999	1.01 (1.00, 1.02)	
65,000+	1.00	
Number of licensed physicians and surgeons		<0.0001
<500	0.89 (0.88, 0.91)	
500–999	0.95 (0.94, 0.96)	
1,000–1,999	0.96 (0.95, 0.97)	
≥2,000	1.00	

Note. Factors in the table adjusted each other through this multivariable ZTNB regression model.

* p-value from F-test for assessing the effect of a factor in multivariable analysis.

Individuals who resided in counties where less than 4% of the population belonged to multiple ethnicities had an approximately 5% lower incidence rate of new prescriber use than those who resided in counties with a more multiethnic mix. Living in counties where 30% or more of the residents did not graduate from high school was associated with an 8% higher incidence rate of new prescriber use than living in other counties. Compared to individuals located in counties where the median annual household income was greater than $65,000, those residing in counties with lower median annual household incomes had either higher or lower incidence rates of new prescriber use, but the effect was negligible (all IRRs were close to 1). The total number of licensed physicians and surgeons in a county was positively, linearly, and independently associated with the number of prescribers that individuals used for prescription opioids. Compared to individuals who lived in counties where at least 2000 licensed physicians and surgeons were available, the adjusted IRRs for those who lived in counties where the number of the licensed physicians and surgeons were <500, 500–999, 1000–1999 were 0.89 (95% CI: 0.88, 0.91), 0.95 (95% CI: 0.94, 0.96) and 0.96 (95% CI: 0.95, 0.97), respectively.

### Factors associated with the incidence rate of pharmacy use

The multivariable analyses showed that all factors were significantly (p <0.0001) associated with the incidence of pharmacy use (Tables 4 and 5). The incidence rate of new pharmacy use also declined across increasing age groups (with the exception of age group 35–44 years, which had a slightly higher incidence rate than that of the 18–34 years age group). Compared to individuals 75 or more years old, individuals who were 18–34 years, 35–44 years, 45–64 years and 65–74 years had 1.75 (95% CI: 1.73, 1.77), 1.80 (95% CI: 1.77, 1.82), 1.45 (95% CI: 1.44, 1.47) and 1.09 (95% CI: 1.08, 1.11) times higher incidence rates of new pharmacy use in one calendar year, respectively, when adjusting for other individual level and county-based factors. Males had a 6 percent lower incidence rate of new pharmacy use than females, after adjusting for other factors (adjusted IRR = 0.94 (95% CI: 0.93, 0.95)). Individuals who used Schedule III opioids alone had a higher incidence rate (adjusted IRR = 1.25 (95% CI: 1.23, 1.26)) of new pharmacy use than those who used Schedule II opioids alone; individuals who used both Schedule II and III opioids had the highest incidence rate (adjusted IRR = 2.83 (95% CI: 2.79, 2.87)). Individuals who received high dose opioid therapy had a 1.83 (95% CI: 1.81, 1.84) times higher incidence rate of new pharmacy use than those who did not. Individuals who lived in counties where less than 4% of the population were comprised of multiple ethnicities had a higher incidence rate of new pharmacy use than those who lived in counties with more multiethnic population. The adjusted IRRs were 1.09 (95% CI: 1.05, 1.13), 1.16 (95% CI: 1.15, 1.17) and 1.03 (95% CI: 1.02, 1.04) when comparing individuals whose residing county had 1–1.9%, 2–2.9%, and 3–3.9% multiethnic population to individuals whose residing county had 4% or more multiethnic population. Compared to individuals who lived in counties where more than 30% residents did not graduate from high school, the adjusted IRRs for individuals living in counties where <10%, 10–19.9%, and 20–29.9% of residents did not graduate from high school were 0.90 (95% CI: 0.87, 0.93), 1.02 (95% CI: 0.99, 1.05), and 1.15 (95% CI: 1.12, 1.18), respectively. As with prescriber use, the effect of the median household income on the new pharmacy use was trivial. Also, the total number of licensed physicians and surgeons in a county was positively, linearly, and independently associated with the number of pharmacies that individuals used for prescription opioids. Compared to individuals who lived in counties where at least 2000 licensed physicians and surgeons were available, the adjusted IRRs for individuals who lived in counties where number of the licensed physicians and surgeons were <500, 500–999, 1000–1999 were 0.68 (95% CI: 0.67, 0.70), 0.73 (95% CI: 0.73, 0.74), and 0.78 (95% CI: 0.77, 0.79), respectively.

**Table 4:** Adjusted incidence rate ratios and 95% confidence intervals (CI) from multivariable ZTNB regression model of individual characteristics for pharmacy use to obtain prescription opioids in California in 2006.

Factor	Adjusted IRR (95% CI)	P-value*
Age (years)		<0.0001
18–34	1.75 (1.73, 1.77)	
35–44	1.80 (1.77, 1.82)	
45–64	1.45 (1.44, 1.47)	
65–74	1.09 (1.08, 1.11)	
75–100	1.00	
Gender		<0.0001
Male	0.94 (0.93, 0.95)	
Female	1.00	
Drug schedule		<0.0001
only Schedule II	1.00	
only Schedule III	1.25 (1.23, 1.26)	
both Schedule II and Schedule III	2.83 (2.79, 2.87)	
Dose type		<0.0001
≤40 mg/day	1.00	
>40 mg/day	1.83 (1.81, 1.84)	

Note. Factors in the table adjusted each other through this multivariable ZTNB regression model.

* p-value from F-test for assessing the effect of a factor in multivariable analysis.

**Table 5:** Adjusted incidence rate ratios and 95% confidence intervals (CI) from multivariable ZTNB regression model of county characteristics for pharmacy use to obtain prescription opioids in California in 2006.

Factor	Adjusted IRR (95% CI)	P-value*
Percent of multiracial population		<0.0001
1–1.9	1.09 (1.05, 1.13)	
2–2.9	1.16 (1.15, 1.17)	
3–3.9	1.03 (1.02, 1.04)	
4+	1.00	
Percent of residents who did not graduate from high school		<0.0001
0.0–9.9	0.90 (0.87, 0.93)	
10.0–19.9	1.02 (0.99, 1.05)	
20.0–29.9	1.15 (1.12, 1.18)	
30.0+	1.00	
Median annual household income		<0.0001
35,000–44,999	0.97 (0.95, 0.99)	
45,000–54,999	0.98 (0.97, 0.99)	
55,000–64,999	1.02 (1.01, 1.03)	
65,000+	1.00	
Number of licensed physicians and surgeons		<0.0001
<500	0.68 (0.67, 0.70)	
500–999	0.73 (0.73, 0.74)	
1,000–1,999	0.78 (0.77, 0.79)	
≥2,000	1.00	

Note. Factors in the table adjusted each other through this multivariable ZTNB regression model.

* p-value from F-test for assessing the effect of a factor in multivariable analysis.

## Discussion

After combining the California PMP data with US census data, we examined the associations of individual-level and county-based factors with use of multiple prescribers and pharmacies for prescription opioids in California during 2006. Younger age and female gender were associated with use of more prescribers and more pharmacies for opioid prescriptions. All county-based factors (ethnicity, educational attainment, median household income and physician availability) were significantly associated with use of multiple prescribers and pharmacies. Among those factors, the physician availability had a positively linear relationship with the number of prescribers and pharmacies that patients used in 2006. Other neighborhood factors such as ethnicity, educational attainment, and median household income had either small or inconsistent association, which may be due to influences from unmeasured factors.

Our findings of the age associations are consistent with the results from studies on illicit drug use and prescription drug abuse. The NSDUH demonstrated that the proportion of residents aged 18 or older who used illicit drugs in the past month declined across increasing age groups, with the peak incidence rate in the 18–25 year group [3]. Similarly, White et al. found that the odds of being involved in prescription opioid abuse declined with age, with a peak in the 18–24 year group [36]. Interestingly, our study revealed that individuals in the 18–34 and 35–44 year groups had similar incidence rates of new prescriber use and new pharmacy use. The similarity between the two age groups may be explained by the high incidence of middle-aged individuals susceptible to chronic pain [9]. Rather than ascribe the behavior of any of these cohorts to abuse, perhaps the supplementary prescribers were dissociated from the primary prescriber, or were associates covering the provider. Moreover, some patients might have visited additional prescribers while seeking pain relief or have used additional pharmacies for legitimate reasons. Unfortunately, this cannot be verified and remains a limitation of the secondary data analysis.

Multiple studies have consistently found that illicit drug use is higher among males [37]. However, findings on gender effects from studies involving prescription opioid abuse have been mixed. For instance, White et al. found that males had 70% higher odds of being prescription opioid abusers than females [36]. However, they included heroin abusers, thereby biasing their results towards a gender distribution among illicit drug users [36]. Another study examined predictors of opioid misuse in individuals with chronic pain and did not find gender a risk factor for prescription opioid misuse [38]. In that study, individuals were derived from referrals within an academic internal medicine practice, which may not be representative of other settings [38]. Hall *et al.* found males were more likely to be involved in prescription drug diversion and females were more likely to use 5 or more prescribers for prescription drug [39]. Simoni-Wastila conducted two studies using data from National Household Survey on Drug Abuse (http://www.ncbi.nlm.nih.gov/pmc/articles/PMC1448242/?tool=pubmed - r1NHSDA) and found females were 1.5–2 times more likely to experience problem use of prescribed opioid analgesics [40], [41]. Moreover, data from substance treatment centers suggests that females were more likely than males to report abuse of any prescription opioid (15.4% females vs. 11.1% males, p<0.001) [42], which is consistent with this study's findings.

Our results revealed that individuals who used both Schedule II and III opioids visited multiple prescribers and multiple pharmacies more often than those who used only one DEA opioid schedule. It is conceivable that these individuals had more severe pain or opioid tolerance, necessitating seeing more prescribers and pharmacies to obtain greater amounts of opioids. Without detailed information on the indication for each prescription, it is not possible to infer a causal relationship between opioid utilization (drug schedule and/or dose type of therapy) and the multiple prescribers and/or pharmacies use – another limitation of a secondary data analysis.

Our data showed that the most robust county-level factor was the physician availability. The positive linear association between the number of available physicians or surgeons in patients' residency county and the number of prescribers and pharmacies that patients used in a year is consistent with another study whereby the number of prescribers per 1,000 people in a county was independently and positively associated with insurance claim rates for opioids [43]. One possible explanation for this finding is that the availability of more prescribers allows legitimate individuals to solicit more physicians in controlling their undertreated pain while simultaneously allowing abusers the opportunity to enlist more prescribers in their efforts to obtain prescription opioids for nefarious purposes. We speculated that a county with higher physician availability may also have higher pharmacy availability, which likewise may explain the positive association between the physician availability and multiple pharmacies use. We were unable to account for the possibility that individuals went to adjacent counties for opioid prescriptions, so the association of the physician availability in a county may have been underestimated. It should be emphasized that, due to the inability to distinguish abusers from legitimate patients, it was not possible for this study to explore the association between physician availability and drug abuse.

Based on previous research on illicit drug use, we hypothesized that regions with more multiethnic residents, a higher proportion of high school dropouts, and lower median household income would be associated with a higher incidence rate of new prescriber use or new pharmacy use. However, the direction of the association between ethnicity, educational attainment, median household income and use of new prescribers or pharmacies was variable and inconsistent. Studies have indicated that illicit drug use is associated with multiple factors, such as an individual's psychological factors [44], socioeconomic status [3], the drug use in individuals' social network [45], and neighborhood social disorder (i.e., low informal social control) [46]–[48] or disadvantage (i.e., low educational attainment and poor economic status) [18], [23], [45]. The same factors may affect multiple prescribers and pharmacies utilization among prescription opioid users. Due to privacy considerations, only limited individual information was available in the California PMP database, precluding more thorough control of confounding and potentially affecting study validity. Future research with more detailed individual information is needed to complement this study. We believe, however, that such unmeasured factors did not substantially affect the association estimates of age, gender, and the number of licensed physicians and surgeons in a county because they should not act as confounders for these variables.

Another limitation of this study was that we had no control over data quality. However, because provision of data is legislatively mandated with penalties for noncompliance, we suspect that the data is of high quality with respect to accuracy and completeness.

Despite the limitations enumerated above, this study is the first to estimate the county-level contextual effect on use of multiple prescribers or pharmacies to obtain pain relieving opiates. This population-based study had a very large sample size, permitting high precision of the incidence rate ratio estimates. Using a mandated collection source avoided underreporting bias, a limitation of self-reported studies. The study population was all outpatient prescription opioid users in California except those obtaining prescription opioids in federal facilities (i.e., facilities associated the Department of Defense or the Department of Veterans Affairs), which minimized the random error of the results.

In summary, our results show that use of multiple prescribers and/or pharmacies to obtain prescription opioids is more common among females and individuals of young to middle ages (18–45 years). Physician availability in a county is positively associated with the number of prescribers or pharmacies that patients used for prescription opioids. Counties with more than 2,000 licensed physicians and surgeons should receive additional scrutiny to prevent multiple provider and pharmacy episodes. Law enforcement authorities would be wise to concentrate efforts on large metropolitan areas. Whether these episodes represent justifiable activities by patients or characterize abuse and diversion cannot be determined from a cursory examination or generalization from epidemiologic data. For the provider, clinical observation, combined with judicious use of PMP data, are necessary to make individualized decisions in the case of a patient seeking opioids.

## Methods

### Study population

This retrospective cohort study used data from the California PDMP, the Controlled Substance Utilization Review and Evaluation System (CURES). This database is operated under the auspices of the California Department of Justice. The system was converted from a paper-based to an electronic controlled substance surveillance system in 1998, about sixty years after California established the first PDMP in the nation. Pharmacists input the drug name, quantity, dosage, and date of the transaction at the point of disbursement into CURES. In addition, the patients' name, date of birth, gender, and address are transmitted, as are the prescriber and pharmacy identities with the DEA registration numbers. To ensure confidentiality and anonymity of the information obtained for this study, the CURES database underwent de-identification as described in one of our previous study [25]. Individuals were included in the study if they had at least one opioid prescription in each of three years, 2005 through 2007 (Table 6). By incorporating three consecutive years, with only prescriptions from 2006 being utilized for data analysis, each patient was assured of one person-year exposure in which they were at risk of using multiple prescribers or using multiple pharmacies. Data was excluded if a prescription was: (1) incomplete with respect to the age, gender and prescriber information; (2) implausible, e.g., a duplicate prescription with the same formulation recorded for the same time period; (3) a commercial transaction whereby the prescription was written for more than 700 pills or 50 patches in a given 30-day period (based on personal communication with the manager of the CURES database in the California Department of Justice); (4) for medications not suggestive of standard delivery systems employed by most chronic pain individuals (e.g., rectal suppositories, intravenous preparations, syrups, solutions, etc.); (5) for use of medication by age groups normally not associated with chronic pain or obtaining medications through office use interactions, i.e., children under the age of 18 and adults over 100 years of age, respectively.

**Table 6:** Type of Opioids Analgesics by Regulation Level.

Drug type	Drug name
Schedule II	Long-Acting Fentanyl
	Long-Acting Levorphanol
	Long-Acting Methadone
	Long-Acting Morphine
	Long-Acting Oxycodone
	Short-Acting Fentanyl
	Short-Acting Hydromorphone
	Short-Acting Meperidine
	Short-Acting Morphine
	Short-Acting Oxycodone
Schedule III	Short-Acting Codeine
	Short-Acting Hydrocodone

The Institutional Review Board of the University of California at Davis and the Veterans Administration Northern California Health Care System Research and Development Committee granted approvals to conduct this research.

### Dependent regression variables

Dependent variables included the number of distinct doctors and the number of distinct pharmacies that an individual used for all his/her opioid prescriptions obtained during January 1, 2006 through December 31, 2006.

### Individual-level factors (age, gender, drug schedule and dose type of opioid therapy)

Each individual's age and gender was tallied. Stratification into five age groups was performed in the manner of previous investigations [26]. The categorization was: 18–34, 35–44, 45–64, 65–74, and 75–100 years of age.

The DEA controlled substance schedule was used to stratify individuals into three groups: those who used only Schedule II opioids, those who used only Schedule III opioids, and those who used both Schedule II and III opioids in calendar year 2006.

The association between individuals' rate of opioid consumption and their use of multiple prescribers and pharmacies was examined using a calculated average daily dose. This quantity was defined as high dose therapy if the average daily dose was greater than 40 mg of morphine-equivalents per day. A low dose was characterized as 40 mg or less of morphine-equivalents per day. This cutoff point was related to the practice of providing a maximum of eight hydrocodone or oxycodone pills in combination with acetaminophen. The limit on so-called “weak opioids”, which in 2006 was 4,000 mg of acetaminophen (that also included 40 mg of an opioid), was designed to prevent toxicity from this otherwise over-the-counter analgesic [27], [28]. More than 40 mg per day of an opioid often involved the addition of a sustained-release preparation with commensurate increases in dosing. The average daily opioid dose was computed using the sum of morphine-equivalents (product of strength, quantity, and an equianalgesic conversion factor [29]) divided by the duration of prescribing. The latter was calculated as the interval between an individual's first and last opioid prescriptions in 2006 plus an estimate of the duration of his/her last prescription utilizing the “last observation carry forward” method [30], [31]. Further details of this methodology were discussed in our previous publication [25].

### County-based factors (ethnicity, education, income and physician availability)

The individual level data was linked to the county-specific survey data obtained from the 2006 US Census Database [32]. We considered four social domains of California counties: ethnicity, educational attainment, economic status and medical resource availability as discussed below:

1. Ethnicity: the percent of the total population in each county in 2006 that self-reported his/her heritage was derived from two or more ethnic groups. This categorization was selected to measure an ethnicity effect inasmuch as the highest rates of past month illicit drug use generally occur among persons reporting themselves to be derived from two or more ethnic groups [33], [34].
2. Educational attainment: the percent of people 25 years and over in each county in 2006 who did not complete high school.
3. Economic status: the median annual household income in each county in 2006.
4. Medical resource availability: the number of licensed physicians and surgeons in each county in 2006, derived from Rand California Community Statistics Database [35].

### Statistical analysis

The two outcomes, number of opioid prescribers and number of pharmacies, were count variables without zero in their range, i.e., all individuals used at least one prescriber and one pharmacy. Therefore, a zero-truncated count model was chosen for the analysis. The initial data exploration showed that the relationship between each of the continuous independent variable and the log of outcome variables was non linear, so all continuous independent variables were polychotomized into categorical variables. Univariable analysis showed the existence of over-dispersion for the outcome variables, so the ZTNB regression model was used as the primary analysis model. PROC NLMIXED in Statistical Analysis System (SAS) was used for the regression analyses and CONTRAST statements in the procedure were used to examine the effect of each factor. Only variables with statistically significant effects in the univariable analysis were included in the final multivariable model. The adjusted incidence rate ratios (adjusted IRR) and 95% confidence intervals (95% CI) from the multivariable model were separately presented for the two outcome variables. All of the analyses were carried out with SAS v9.2 (SAS Institute Inc., Cary, NC, USA).
